# Characterization of Novel Pullulanase Type I from Newly Isolated *Bacillus cereus* Strain NP9: Potential Additive for Laundry Detergent Formulations

**DOI:** 10.1007/s12010-025-05286-1

**Published:** 2025-06-18

**Authors:** Nihan Arabaci

**Affiliations:** Biology Department, Arts and Sciences Faculty, Cukurova University, Adana, Turkey

**Keywords:** Pullulanase type I, Bacillus, Laundry detergents, TLC, HPLC, Native-PAGE

## Abstract

This study aimed to produce a pullulanase that can be utilized as an additive in detergent formulations. A newly isolated *Bacillus cereus* strain NP9 exhibited the highest pullulanase activity and was selected for production. The optimum conditions for crude NP9 pullulanase were a pH of 7.0 and a temperature of 40 °C. It maintained stability at high rates within the pH range of 5.0 to 11.0 and temperatures between 25 and 65 °C. The molecular weight of the enzyme was determined to be approximately 170 kDa via native-PAGE. Thin-layer chromatography and high-performance liquid chromatography analyses indicated that NP9 pullulanase converted pullulan and starch substrates into maltotriose units (pullulanase type I). The enzyme exhibited moderate activity with certain metal ions and was not Ca^2+^-dependent. The inhibition of the enzyme by EDTA, EGTA, and 1,10-phenanthroline indicated it is a metalloenzyme. The enzyme moderately retained activity when exposed to non-ionic detergents such as Triton X-100, Tween 20, and Tween 80. It demonstrated high compatibility (90%) with the commercial detergent “Peros.” Wash performance analyses showed that the NP9 pullulanase and commercial detergent mixture removed starchy stains more effectively than washing with commercial detergent alone. In conclusion, NP9 pullulanase exhibited favorable properties, making it a potential candidate for the laundry detergent industry.

## Introduction

Starch is among the most abundant polymers in nature. It has become a valuable raw material that can be converted into usable products by various enzymes across diverse industrial fields [1, 2]. Hydrolysis of starch, which consists of amylose (α−1,4 glycosidic linkages) and amylopectin (α−1,4 and α−1,6 glycosidic linkages), involves debranching enzymes known as exoamylases, endoamylases, transferases, and starch processing enzymes [2, 3]. Most of these enzymes are categorized within glycosyl hydrolases family 13 (http://www.cazy.org/) [4]. The most effective enzymes in this category, α-amylases and pullulanases, constitute approximately 30% of the global enzyme market [3].

Pullulanases (EC 3.2.1.41) are debranching enzymes that, together with amylolytic enzymes, hydrolyze α−1,6 and α−1,4 glycosidic bonds in starch, pullulan, and related polysaccharides [3, 5]. They are categorized into five groups based on reaction end products and substrate specificity [2, 6]:

(i) Pullulanase type I (hydrolyzes α−1,6 bonds in starch and pullulan to maltotriose).<div class="NodiCopyInline">Pullulanase type I (hydrolyzes α−1,6 bonds in starch and pullulan to maltotriose).</div>
(ii) Pullulanase type II (amylopullulanase) (hydrolyzes both α−1,6 and α−1,4 bonds in starch and α−1,6 bonds in pullulan).
(iii) Pullulan hydrolase type I (neopullulanase) (hydrolyzes α−1,4 glycosidic bonds in pullulan to panose).
(iv) Pullulan hydrolase type II (isopullulanase) (hydrolyzes α−1,4 glycosidic bonds in pullulan to isopanose).
(v) Pullulan hydrolase type III (hydrolyzes α−1,6 and α−1,4 bonds in pullulan to a mixture of maltose, maltotriose, and panose)

In particular, pullulanase type I can completely hydrolyze starch-based polysaccharides in conjunction with amylases. This accelerates the saccharification of starch, even with low levels of amylase, shortens the reaction time, and results in higher yields when higher substrate concentrations are present [7].

Pullulanases are produced by bacteria, fungi, and some higher plants [8]. Due to their usability in many industrial areas, there is a great interest and tendency towards bacteria, which are the potential producers of pullulanases with various chemical properties, such as being thermostable, pH-stable, and inhibitor-tolerant [9]. Additionally, their ease of production on a large scale, their stable properties during processes, and their relatively low production costs make them a preferred source in industrial applications [4]. Especially, the genus Bacillus is an important group of bacteria frequently preferred in industrial enzyme production, can synthesize extracellular enzymes in high yields, and is generally considered safe (GRAS)[5, 10]. Various Bacillus pullulanases are commercially available on the market [11]: Optimax® L-1000 (DuPont Genencor® Science, from *B. deramificans*), Promozyme® D2 (Novozyme, from *B. acidopullulyticus*), PUL2 (Sunsonzymes, from *B. subtilis*), and PU-799 (Boli Bioproducts, from *B. licheniformis*) [3, 12].

Microbial pullulanases are used in a wide variety of industries: the starch processing industry (in the manufacturing of glucose, maltotriose, maltotetraose, panose, isopanose, and fructose syrups), production of high maltose corn syrup (for high-quality candy/ice cream and intravenous feeding), production of high-fructose corn syrup (for diabetic food formulation), production of cyclodextrins (for cholesterol-free products), brewing industry (for low-calorie beer), production of resistant starch, beverage industry (for clarification of fruit juices), baking industry (as an anti-staling agent), starch saccharification process, detergent industry (as an additive for formulations), bioethanol production, preparation of dental plaque control agents with resistant starch [2, 3, 7, 13].

Microbial enzymes help to increase the cleaning performance of the laundry detergents, dishwashing liquids, industrial washing detergent/institutional cleaning products, and various household cleaners in which they are added to the formulations, to improve their environmental impact and to make them more economical and green products [14, 15]. These enzymes should have activity at low temperatures, high thermostability, stability in alkaline conditions, and stability with other components of the detergent. The ability of enzyme-based detergents to remove stains at moderate temperatures makes them more environmentally friendly and economical. It also protects and repairs laundry fabrics [14, 16, 17]. Microbial enzymes added to detergents replace phosphates and various bleaching chemicals, eliminating their harmful effects. This not only facilitates the removal of tough stains but also positively contributes to public and environmental health [18].

In starch-based industrial processes, thermal and acid-tolerant pullulanases are generally preferred. However, considering their environmental friendliness, ability to reduce energy consumption, and cost-effectiveness, pullulanases (particularly pullulanase type I) that show catalytic activity at moderate temperatures should be studied frequently and included more in industrial processes [2, 19]. In addition, alkali-tolerant and detergent-resistant pullulanases are more common in the detergent industry. Studies on pullulanases that can work under moderate conditions of laundry are limited in the literature [20]. But recently, alkali-stable, detergent-resistant, and mesophilic pullulanases type I, active at moderate temperatures, have also been reported [2]. In the literature review, we did not encounter a study on moderate pullulanase type I among the studies conducted with pullulanases that can be used as detergent additives. In this context, this situation allowed us to evaluate our study as original and pioneering research.

This study aimed to isolate a bacterial pullulanase type I producer strain, obtain and produce the enzyme, perform biochemical characterization, and determine its potential as a detergent additive. For these purposes, *B. cereus* strain NP9 isolated from soil was selected as the best pullulanase producer among the screened bacterial isolates. Then, the NP9 pullulanase was isolated as a raw enzyme. The enzyme was investigated for its exceptional biochemical properties and usability in the detergent industry.

## Materials and Methods

### Isolation of Bacteria

One gram of soil sample was added to the 50 mL Luria–Bertani media (LB) (pH 7.0), including (g/L) yeast extract, 5; tryptone, 10; and NaCl, 10 [21]. To isolate endospore-forming bacteria, the media were heat-shocked at 80 °C for 10 min and incubated at 37 °C and 150 rpm for 24 h [22]. The samples were then serially diluted. One hundred microliters of each diluted sample was spread on LB agar (pH 7.0) and incubated at 37 °C overnight. The pure bacterial isolates obtained were transferred to LB agar slants and stored for later use [9]. Morphological characteristics (shape, color, Gram’s staining) of the isolated strain cell and colony were examined on nutrient agar plates and a microscope.

### Screening of Bacterial Isolates for Pullulanase Activity

Pullulanase production screening of isolated strains was performed on a medium containing (g/L) yeast, 3; tryptone, 5; MgSO_4_, 0.1; K_2_HPO_4_, 0.5; NaCl, 5; pullulan, 5; and agar, 15 (pH 7.0) [23]. The agar plates cultivated with strains were incubated at 40 °C for 48 h. To determine the pullulanase activity, ethanol was poured onto the produced colonies, and the plate was left at − 20 °C for 2 h. Colonies surrounded by clear hydrolysis zones were evaluated as pullulanase positive [24]. Then, the best pullulanase producer strain was selected.

### Molecular Identification of Bacterial Strain

The selected bacterium was identified by a 16S rRNA sequencing molecular test (BM Laboratory Systems Company, Ankara, Türkiye). The procedure followed by the company is as follows: DNA isolation was performed using the “EurX GeneMATRIX Bacterial & Yeast DNA isolation kit (Poland).” For determination of the species, 27 F (5′-AGA GTT TGATCMTGG CTC AG-3′) and 1492R (5′-TAC GGY TAC CTT GTT ACG ACTT-3′) universal primers were used to amplify the targeted region of the gene. In PCR analysis, the initial denaturation was performed at 95 °C for 5 min. Thereafter, 30 cycles were performed under the following conditions: denaturation, 45 s at 95 °C; annealing, 45 s at 57 °C; extension, 60 s at 72 °C. The final extension step was carried out at 72 °C for 5 min. BigDye Terminator v3.1 Cycle Sequencing Kit (Applied Biosystems, Foster City, CA) and ABI 3730XL Sanger sequencer (Applied Biosystems, Foster City, CA) were used for Sanger sequencing. The BLAST program searched for homologous sequences in the NCBI database. The maximum likelihood method and the Tamura-Nei model were used to infer the evolutionary history [25]. MEGA11 software was preferred during the evolutionary analyses [26].

### Production of Pullulanase

Five percent (v/v) of the strain inoculum previously produced in LB broth (40 °C, 180 rpm) was inoculated into Erlenmeyer flasks containing pullulan medium (g/L): yeast, 3; tryptone, 5; NaCl, 5; K_2_HPO_4_, 0.5; MgSO_4_, 0.1; pullulan, 5 (pH 7.0) [23]. The culture flasks were incubated at 40 °C for 48 h at 180 rpm in an orbital shaker [27]. After incubation, the culture broths were centrifuged at 7500 rpm for 20 min at 4 °C. The supernatant was collected for further experiments as a crude enzyme source [9].

### Enzyme Activity Assay and Protein Determination

To evaluate the standard enzyme activity, the 3,5-dinitrosalicylic acid (DNS) method was used [28]. 0.5 mL of 1% pullulan prepared in 0.2 M sodium phosphate buffer (pH 7.0) and 0.5 mL of the enzyme were mixed and incubated at 40 °C for 60 min [29]. After the incubation, 1 mL of DNS reagent was added to the reaction mixture to terminate the reaction. The mixture was incubated at 100 °C for 10–15 min for color development and then cooled. The absorbance was measured at 540 nm [30]. One unit of enzyme activity was defined as the amount of enzyme releasing 1 µmol of reducing sugar (glucose) per minute under standard assay conditions. Triplicate measurements were made, and the activity value was determined by taking their averages. To determine the protein content of each sample, the Bradford method was performed using a bovine serum albumin standard curve [31]. The specific activity is the number of enzyme activity units per milligram of enzyme protein [9].

### Molecular Weight Determination and Zymogram Analysis

The molecular mass of pullulanase was detected by native-PAGE (10%) containing 1% (w/v) pullulan as a specific substrate [32]. The molecular weight calculation of the enzyme was made by comparing it with non-denaturing protein molecular weight standards. The molecular mass of the enzyme was calculated by comparing it with non-denaturing protein molecular weight standards (Sigma-Aldrich, MWND500). A 20-mA constant current was applied during electrophoresis. The gel was kept in Congo red solution (1 g/L) for 30 min and then in iodine solution (iodine 5 g/L, KI 0.5 g/L) for 15 min at room temperature.

### Effect of pH and Temperature on Pullulanase Activity and Stability

Standard activity analyses were performed using various pH buffers (0.2 M) (citrate–phosphate, pH 4.0 and 5.0; sodium phosphate, pH 6.0–8.0; glycine–NaOH, pH 9.0 and 10.0; sodium carbonate-bicarbonate, pH 11.0; disodium hydrogen phosphate-trisodium phosphate, pH 12.0), and the optimum pH was determined at 40 °C for 60 min [13, 33]. One percent pullulan substrate was added to each pH buffer solution to determine pullulanase activity. The pullulanase was pre-incubated in substrate-free pH buffers (4.0–12.0) at 25 °C for 24 h for pH stability determination. The standard enzyme procedure tested the residual activities [34].

To determine the optimal temperature, the reactions were performed at temperatures ranging from 25 to 70 °C at pH 7.0 for 60 min [23]. For the thermal stability analysis, NP9 pullulanase was pre-incubated at different temperatures (25–70 °C) for 60 min in sodium phosphate (pH 7.0) without the substrate [4, 23], and the residual activities were assayed using the standard method already described.

### Effects of Some Chemicals on Pullulanase Stability

To study the effects of certain chemicals on NP9 pullulanase activity, the enzyme activity was assayed with different concentrations of metal chloride salts (CaCl_2_, AgNO_3_, BaCl_2_, CoCl_2_, FeCl_2_, CuCl_2_, NiCl_2_, MgCl_2_, HgCl_2_, ZnCl_2_, MnCl_2_), inhibitors (iodoacetamide, 1,10-phenanthroline, phenylmethylsulfonyl fluoride (PMSF), ethylenediaminetetraacetic acid (EDTA), ethylene glycol tetraacetic acid (EGTA), urea, β-mercaptoethanol, *N*-alpha-p-tosyl-L-lysine chloromethyl ketone (TLCK)), detergents/anionic-non-ionic surfactants (sodium dodecyl sulfate (SDS), Triton X-100, Tween 20, Tween 80, cetyltrimethylammonium bromide (CTAB)), an oxidizing agent (H_2_O_2_), organic solvents (ethanol, *n*-butanol, methanol, isopropanol, acetone, PEG6000, PEG8000, glycerol), and different NaCl concentrations at 40 °C for 60 min [7, 9, 13, 35, 36]. After the pre-incubation, residual activities were measured using the standard method. Control was considered enzyme activity determined by the standard assay (100%).

### Hydrolytic Properties of NP9 Pullulanase: TLC and HPLC

The hydrolytic properties of pullulanase were mainly analyzed by HPLC (high-performance liquid chromatography) and TLC (thin-layer chromatography). To perform HPLC and TLC analysis, NP9 pullulanase and 0.2 M sodium phosphate solution at pH 7.0 (separately, containing 1% pullulan and starch) were incubated at a 1:1 concentration at 40 °C for 60 min. The TLC plate (TLC_F254_ plates, 20 × 20 cm^2^, Merck) was spotted with the reaction products (15 µL) and the standard carbohydrates (5 µL) (glucose, maltose, maltotriose, 1% (w/v), Merck, Darmstadt, Germany) and placed in a chamber containing a mobile phase (*n*-butanol/acetic acid/distilled water (3:1:1, v/v/v)) [36]. Then, the chromatography process was carried out for 12 h. After the period, the plate was dried at room temperature for 60 min, and the reducing sugars were detected by spraying it with a dye solution (1% aniline and 1% diphenylamine were dissolved in acetone, and then, orthophosphoric acid was added to make up 10% of the total solution volume) [13]. The TLC plate was air-dried again at room temperature for 60 min and heated at 120 °C in a hot-air oven for 30 min until spots appeared [4, 17].

For HPLC, the final products obtained from the 60-min pullulan and starch hydrolysis reaction were diluted tenfold with ultrapure water, and then, the mixtures were centrifuged at 6000 rpm, 4 °C for 10 min. The supernatants were passed through the nylon membrane filter (0.45 µm) before injection into the HPLC system (Shimadzu, Japan). The separation was performed with a Concise-Coregel/Ion 300 column. The mobile phase was H_2_SO_4_ (5 mM, 0.4 mL min^−1^). The chromatography conditions, detector, wavelength, injection volume, and temperature, were Photo Diode Array (PDA)/RID, 210–244 nm, 20 µL, and 50 °C, respectively. The high-purity standard (maltotriose) was used to quantify and identify the sugar peaks. The peaks were defined by comparing spectral data and the standards’ retention times [37].

### Compatibility of Pullulanase with Some Commercial Laundry Detergents

OMO (Unilever, England), Peros (Beyaz Kağıt, Türkiye), Perwoll (Henkel, Germany), and Persil (Henkel, Germany) were used to determine the compatibility of pullulanase with commercial liquid laundry detergents. Before the stability test, the detergent solutions prepared at 1% (w/v) concentrations were heated to 100 °C for 30 min to inactivate the endogenous enzymatic activity [35, 38]. After incubating the enzyme and detergents at 40 °C for 60 min, the standard activity method was used to determine the residual activity. The activity of pullulanase without any detergent was evaluated as a control (100%) [36].

### Wash Performance of NP9 Pullulanase

White cotton clothes (4 × 4 cm^2^) were stained with chocolate, ketchup, apricot puree, and beet juice and dried overnight at room temperature. In the washing medium, the final volume of NP9 pullulanase is 5%, and the final volume of the heat-inactivated commercial liquid laundry detergent “Peros” is 1%. Wash performance analysis was carried out as follows: (A) stained cloth + 20 mL tap water (control); (B) stained cloth + 0.2 mL liquid detergent (1%) + 19.8 mL tap water; (C) stained cloth + 1 mL enzyme (5%) + 19 mL tap water; (D) stained cloth + 1 mL enzyme (5%) + 0.2 mL liquid detergent (1%) + 18.8 mL tap water. These washing media prepared in beakers were incubated at 40 °C and 150 rpm for 60 min. Then, the cotton clothes were gently rinsed with tap water, air-dried, and visually evaluated. The untreated clothes stained with chocolate, ketchup, apricot puree, and beet juice were taken as controls. Moreover, standard enzyme activity analysis was carried out with the washing water (containing the hydrolysis products of carbohydrate-based stain) remaining in the beaker after the wash performance analysis [36, 38].

## Results and Discussion

### Characterization of Pullulanase‑Producing Strain NP9

**Fig. 1:**
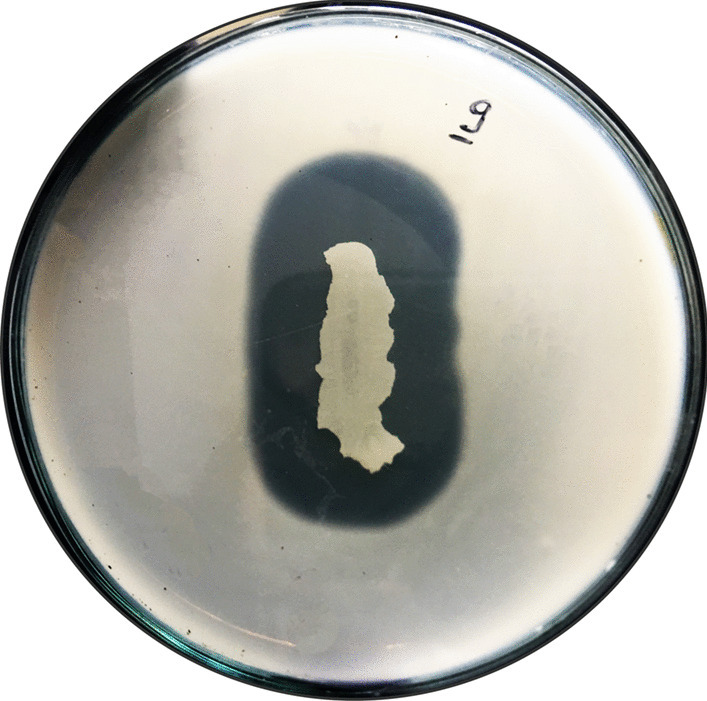
Hydrolysis activity circle of Bacillus cereus strain NP9 pullulanase on pullulan agar

**Fig. 2:**
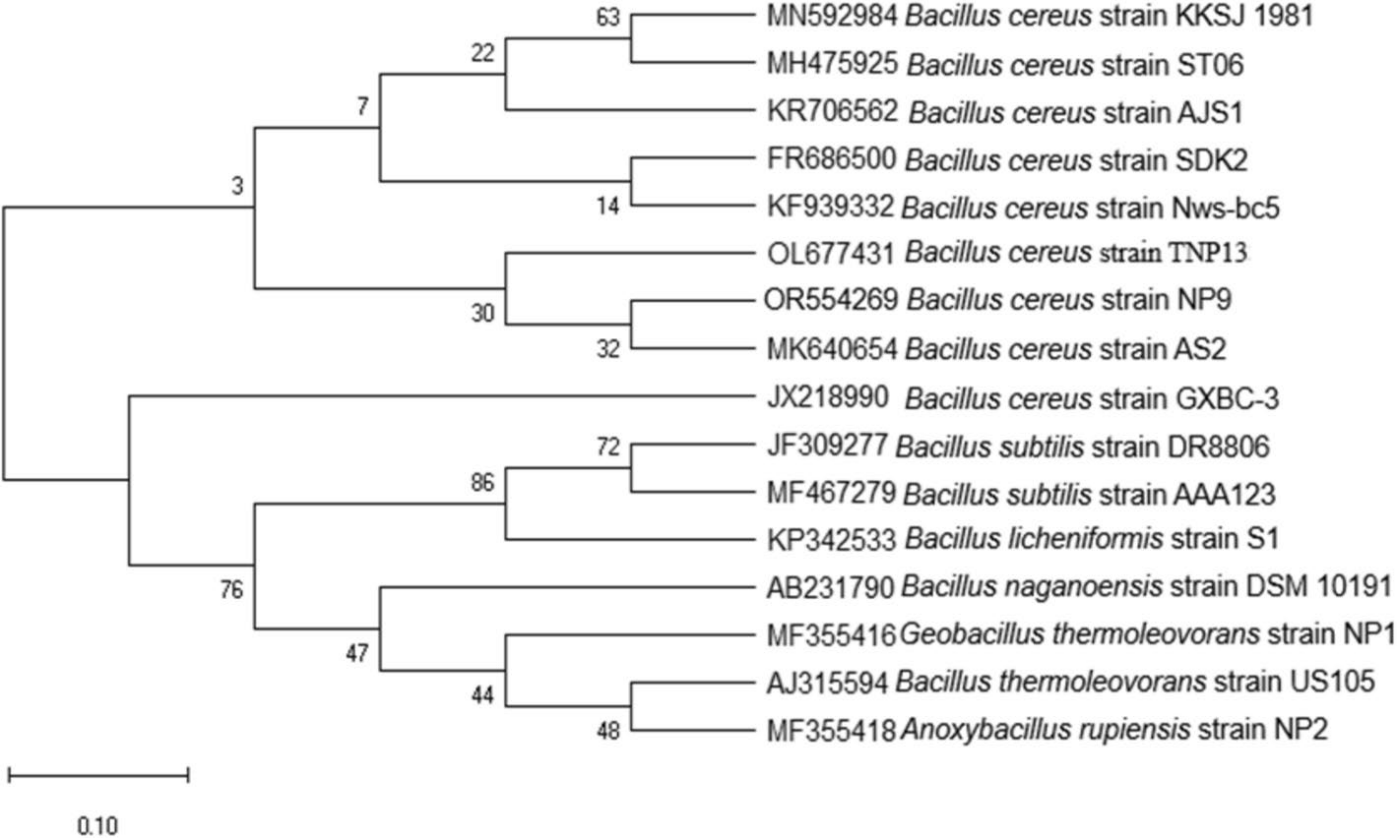
Phylogenetic tree of *Bacillus cereus* strain NP9

The “NP9” strain was selected from approximately 50 bacterial colonies isolated from the soil samples and prepared as a pure culture. The strain demonstrated pullulanase activity when cultivated on the pullulanase screening medium. A significant hydrolysis halo zone appeared around the colony when the plate was treated with ethanol at 40 °C and pH 7.0 after 48 h **(**Fig. 1**)**. The novel strain NP9 was a gram-positive, endospore-forming, rod-shaped, aerobic, and catalase-positive bacterium. Its 16S rRNA gene sequence, with a length of 953 bp, showed significant homology with *Bacillus cereus* by BLAST, and the strain was identified as “*Bacillus cereus* strain NP9.” The sequence registered in the NCBI GenBank has an accession number of OR554269.1 (https://www.ncbi.nlm.nih.gov/nuccore/OR554269.1). The phylogenetic tree of the strain NP9 was constructed by MEGA11 **(**Fig. 2**)**. When reviewing the literature, various studies conducted over many years show that pullulanases are predominantly produced by bacterial strains of the Bacillus genus. Additionally, they can be obtained from diverse microbial sources [9, 29, 39, 40].

### Production of Pullulanase

The strain NP9 was cultured in pH 7.0 pullulan broth at 40 °C and 180 rpm for 48 h. The supernatant, the crude enzyme source, was obtained after the culture medium was centrifuged.

In some studies conducted with various Bacillus strains, it has been reported that the pullulanases obtained were produced in 48, 72, and 120 h [27, 41, 42]. It has been determined that the production time of the NP9 pullulanase is quite short compared to other studies in the literature. The fact that the bacteria produce the enzyme in a short time will provide advantages for industrial processes in terms of preventing contamination, reducing the cost of enzyme production, and saving time by accelerating the process period [9]. The specific activity of NP9 pullulanase was 0.2043 U/mg, while the protein amount of the enzyme was 16.2 mg/mL.

### Zymogram Analysis of NP9 Pullulanase (Native-PAGE)

**Fig. 3:**
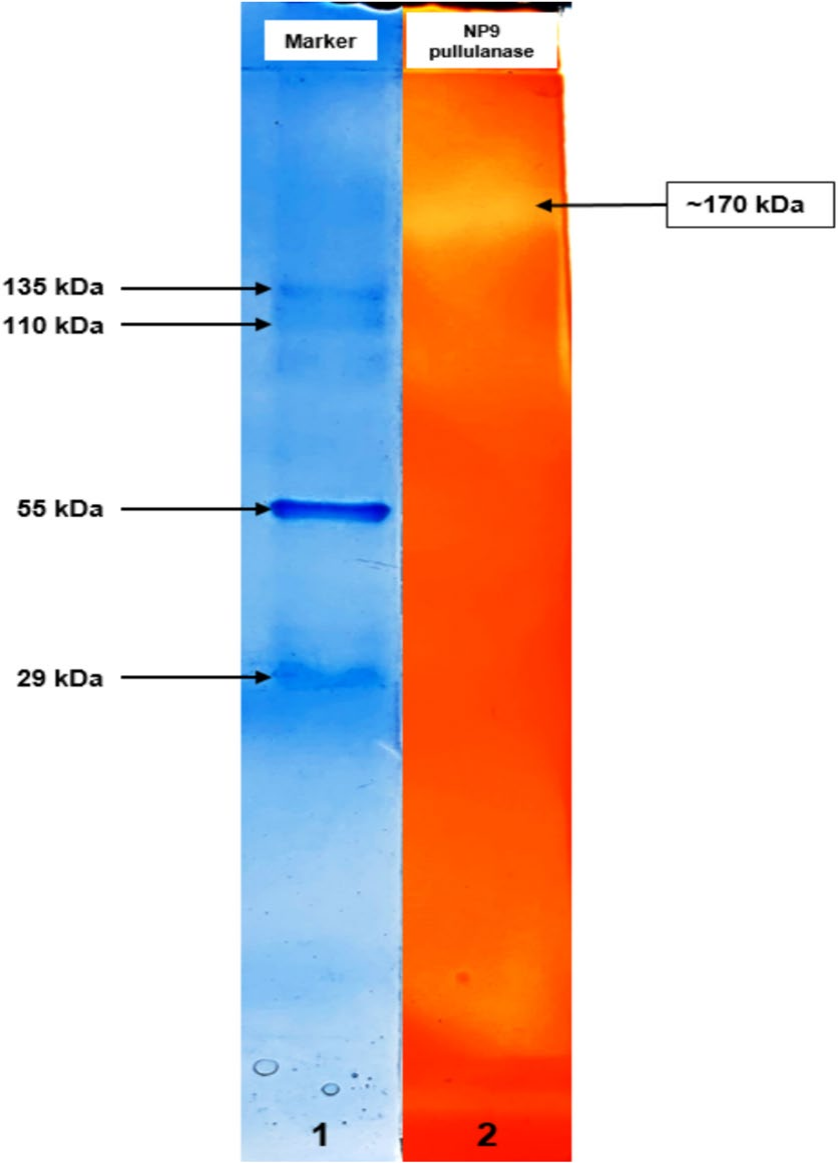
Zymogram analysis of NP9 pullulanase on native-PAGE gel (10%) containing pullulan (1%). Lane 1, marker (Sigma-Aldrich, MWND500); lane 2, activity band of crude NP9 pullulanase

In zymogram, a single activity band of NP9 pullulanase with a molecular weight of approximately 170 kDa was detected on a 10% native-PAGE gel with 1% pullulan. A clear activity band appeared on the red background of the gel stained with Congo red and iodine solution **(**Fig. 3**)**. Zymogram helped to determine NP9 pullulanase activity. Although SDS-PAGE is a sufficient analysis for determining the molecular weight of the protein, performing native gel electrophoresis is a more comprehensive analysis for determining further structural properties of the protein in its native state [43]. Various studies have reported different molecular weights of pullulanases isolated from Bacillus strains: *Bacillus* sp. S-1 (140 kDa) [44], *Bacillus* sp. KSM-1876 (120 kDa) [40], *Bacillus* sp. CICIM 263 (101 kDa) [45], *B. cereus* ATCC 14579 (95 kDA) [46], *B. megaterium* Y103 (83.2 kDa) [20]**,** *B. cereus* Nws-bc5 (81.4 kDa) [23], *B. safensis* (42 kDa) [9], *B. holodurans* (37 kDa) [42].

### Effects of pH and Temperature on NP9 Pullulanase Activity and Stability

**Fig. 4:**
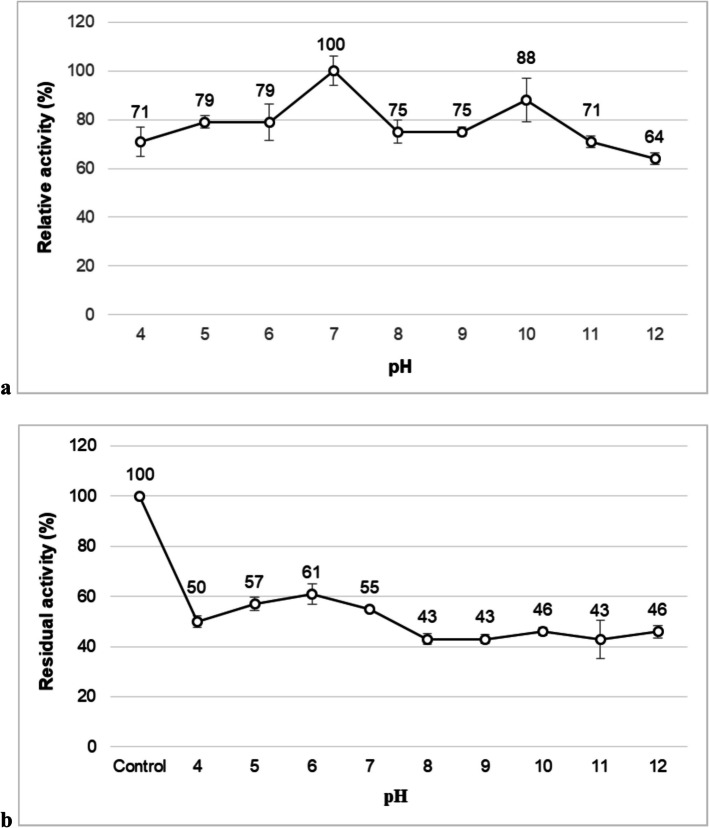
Effect of pH on activity (**a**) and stability (**b**) of NP9 pullulanase. Each value is the average of three replicates, and standard error bars are included

Figure 4 shows the effect of pH on NP9 pullulanase activity. The optimum activity of NP9 pullulanase was obtained at pH 7.0 **(**Fig. 4a**)**. However, the enzyme exhibited more than 75% relative activity in the pH range of 5.0–11.0, showing quite good activity in a wide pH range. The NP9 pullulanase showed maximum stability at pH 6.0, with 61% of its initial activity, while at pH 7.0, the remaining activity of 57% was detected. The enzyme was stable at pH 8.0–12.0 with a residual activity of 45% recorded at 60-min incubation **(**Fig. 4b**)**. Similar studies have been reported in the literature. Pullulanase obtained from *B. safensis* demonstrated its optimal activity at pH 7.0 and remained stable at 91% and 94% in the pH range of 5.0–6.0, respectively. It exhibited 40% stability in the pH range of 9.0–11.0 [9]. *B. cereus* ATCC 14579 pullulanase also exhibited its optimal activity at pH 7.0 and retained almost all of its initial activity in the pH range of 6.0–8.0 [46]. Novel pullulanase, produced by *Paenibacillus lautus* DSM 3035, is an enzyme that shows its maximum specific activity at pH 7.0 and remains stable at almost 50% in the pH range of 6.5–9.0 [47]. Besides these, there are also bacterial pullulanases that show their maximal activity at various pH values, such as pH 6.0 [1], 6.5 [48], and 9.0 [7]. Pullulanase from *Geobacillus kaustophilus* DSM7263 showed the highest stability at pH 6.0 and retained more than 70% of its initial activity in the pH range of 5.0–9.0 [49].

Pullulanase type I from *Paenibacillus barengoltzii* retained approximately 80% of its initial activity for 30 min in the pH range 5.5–10.5 [48]. NP9 pullulanase’s high activity over a wide alkaline pH range and moderate stability make it applicable in various industrial areas, especially in the detergent industry as a detergent additive [38].

**Fig. 5:**
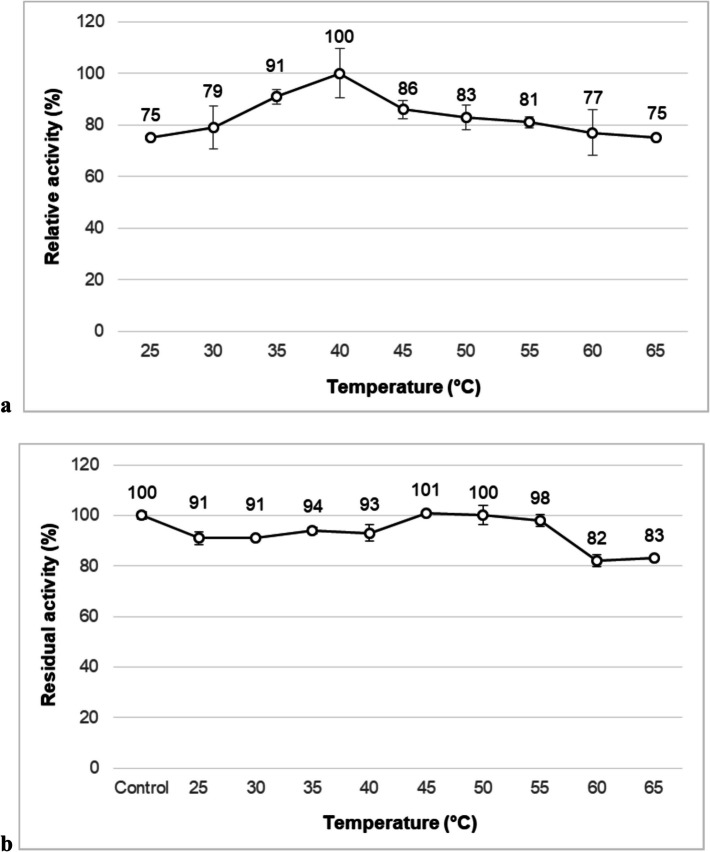
Effect of temperature on activity **(a)** and stability **(b)** of NP9 pullulanase. Each value is the average of three replicates, and standard error bars are included

When the effect of temperature on NP9 pullulanase activity was examined, the optimum temperature was determined as 40 °C. In addition, 78% and 87% of relative activities were observed at 35 and 45 °C, respectively. Moreover, 75% enzyme activity was recorded even at 65 °C **(**Fig. 5a**)**. It has been observed that the enzyme is most stable at 45 and 50 °C, preserving all of its activity and remaining stable at an average rate of 95% in the range of 25–55 °C after 60-min incubation. Furthermore, it retained 82% and 83% residual activities even at 60 and 65 °C, respectively, indicating that it has thermostable/thermotolerant character **(**Fig. 5b**)**. Thermotolerant and thermostable pullulanases are preferred, especially in starch-based industrial processes, as they increase the solubility of starch and similar oligosaccharides, prevent microbial contamination, and reduce the reaction time [9]. In some studies, pullulanases that exhibit activity at the same and different optimal temperature values as NP9 pullulanase have been reported. Pullulanase obtained from *Paenibacillus lautus* DSM 3035, which lost stability sharply above 45 °C [47], and pullulanase type I produced by *B. megaterium* Y103, which maintained its initial activity below 40 °C [20], showed optimum activities at 40 °C, similar to pullulanase from *B. cereus* strain NP9. According to Weiand colleagues [50], most pullulanase type I enzymes in the literature exhibit thermophilic or mesophilic properties. Besides these, various bacterial pullulanases have been reported, which demonstrated their maximum activity at 35 °C [48], 37 °C [41], 50 °C [7], 65 °C [11], and 70 °C [45]. PulASK pullulanase type I from *Anoxybacillus* sp. SK3-4 was stable between 30 and 60 °C and retained 50% of its activity at 60 °C for 9 h [51]. PulGK pullulanase retained more than 70% of its initial activity between 55 and 70 °C but lost stability above 70 °C and completely lost its activity after 24 h at 75 °C [49]. PUL_BC_ pullulanase type I showed more than 80% of its initial activity at 20–40 °C, with the highest stability value at 35 °C. When the enzyme was incubated for 60 min, its stability gradually decreased above 50 °C and was completely lost at 70 °C [23].

### Effects of Chemicals on NP9 Pullulanase

The effects of some metal ions, surfactants, oxidizing agents, inhibitors, and organic solvents were assayed to check whether they increased or decreased the activity of NP9 pullulanase (Table 1). The effects of two concentrations of each chemical on the enzyme were tested. Some chemicals slightly inhibited the enzymatic activity in a concentration-dependent manner.

**Table 1:** Effects of certain chemicals on NP9 pullulanase

Chemicals	Concentrations	Residual activity (% ± SD*)
Control	-	100
**Metal ions**		
Ag^+^	5 mM 10 mM	61 ± 9.85 52 ± 0
Ba^2+^	5 mM 10 mM	32 ± 8.45 41 ± 0.21
Ca^2+^	5 mM 10 mM	56 ± 1.00 51 ± 2.62
Co^2+^	5 mM 10 mM	43 ± 2.83 49 ± 1.02
Cu^2+^	5 mM 10 mM	31 ± 6.29 29 ± 2.86
Fe^3+^	5 mM 10 mM	51 ± 3.93 41 ± 3.93
Hg^2+^	5 mM 10 mM	37 ± 2.11 3 ± 1.32
Ni^2+^	5 mM 10 mM	48 ± 1.00 45 ± 4.38
Mg^2+^	5 mM 10 mM	56 ± 4.54 64 ± 1.35
Mn^2+^	5 mM 10 mM	34 ± 2.86 42 ± 1.88
Zn^2+^	5 mM 10 mM	51 ± 9.88 42 ± 3.74
**Inhibitors/oxidizing agent**		
Iodoacetamide	1 mM 5 mM	47 ± 2.67 20 ± 2.35
1,10-Phenanthroline	1 mM 5 mM	42 ± 3.41 59 ± 6.82
EDTA	1 mM 5 mM	35 ± 4.24 46 ± 4.40
EGTA	1 mM 5 mM	70 ± 8.27 47 ± 6.13
PMSF	1 mM 5 mM	54 ± 2.42 78 ± 0.92
Urea	5 mM 10 mM	40 ± 0.03 48 ± 0
TLCK	1 mM 2 mM	40 ± 0.07 38 ± 1.17
β-Mercaptoethanol	0.02% 0.05%	53 ± 2.85 11 ± 3.59
H_2_O_2_	5% 10%	1 ± 0.05 1 ± 0.04
**Surfactants**		
Tween-20	0.5% 1%	51 ± 2.70 43 ± 0.34
Tween-80	0.5% 1%	62 ± 6.20 40 ± 6.95
Triton X-100	0.5% 1%	46 ± 2.72 44 ± 1.53
SDS	0.5% 1%	38 ± 0 34 ± 0.68
CTAB	0.5% 1%	44 ± 4.92 29 ± 3.20
**Organic solvents**		
Ethanol	10% 20%	48 ± 3.49 37 ± 1.68
* n*-Butanol	10% 20%	37 ± 0 42 ± 0
Methanol	10% 20%	46 ± 7.69 42 ± 1.88
Isopropanol	10% 20%	54 ± 1.28 64 ± 0
Acetone	10% 20%	66 ± 4.36 55 ± 5.38
PEG6000	1% 5%	41 ± 0.63 35 ± 1.23
PEG8000	1% 5%	53 ± 3.80 32 ± 2.10
Glycerol	5% 10%	31 ± 1.10 31 ± 0

*Each value is the average of three replicates, and standard errors are added

NP9 pullulanase generally retained half or more of its activity with Ag^+^, Ca^2+^, Fe^3+^, Mg^2+^, Ni^2+^, and Zn^2+^, while its activity decreased to approximately 40% in the presence of Ba^2+^, Co^2+^, Cu^2+^, Hg^2+^, and Mn^2+^. Similar results were also obtained in other pullulanase studies [4, 9, 20, 49]. In addition, the fact that the activity of NP9 pullulanase does not increase in the presence of Ca^2+^ shows that the enzyme is not Ca^2+^-dependent. Similar results were obtained in certain pullulanase studies [38, 52]. The non-Ca^2+^ dependency means that there will be no need for the removal of Ca^2+^ at the end of the process, which causes extra cost, in the starch and food industry, where this enzyme has the potential to be used [53, 54].

The inhibitor-enzyme interaction gives information about the enzyme’s need for cofactors and its active site’s structure [55]. NP9 pullulanase lost some activity in the presence of chelating reagents such as EDTA, EGTA, and 1,10-phenanthroline, showing that the enzyme exhibits the characteristics of a metalloenzyme. They chelate divalent cations and prevent enzyme activity by removing metal ions required for the activity [8, 56]. This suggests that metal is an important factor affecting the conformational stability of the enzyme [35]. It is noteworthy that EDTA prevents the activity of cation-dependent enzymes [57]**.** Accordingly, NP9 pullulanase needs metal cofactor(s) for its activity. Similar to the findings obtained in our research, there are studies conducted with different pullulanases. Dakhmouche Djekrif and colleagues [36] reported that EDTA inhibited ABS7 pullulanase activity by 35%. In the study of Ling and co-workers [29]**,** it was reported that pullulanase of *B. cereus* H1.5 strain lost 92% of its activity when exposed to 10 mM EDTA. Five-millimolar EDTA also inhibited *B. subtilis* pullulanase type I [49]. In contrast, pullulanase type I from *Paenibacillus barengoltzii* retained its initial activity with 1 mM EDTA [4]. PMSF blocks the serine residues in the enzyme’s active site and inhibits the activity [24]. The 46% inhibition of NP9 pullulanase with 1 mM PMSF means that the enzyme has serine residues in its active site and is of serine character. In a study by Bedan [58], the pullulanase maintained about 78.4% and 73.6% of its initial activity at 0.1 mM and 1 mM PMSF concentrations. A pullulanase type I of *B. cereus* Nws-bc5 was inhibited by 35% by 10 mM PMSF [23]. The inhibition of NP9 pullulanase by β-mercaptoethanol and iodoacetamide shows that the thiol group is involved in the enzyme activity and affects the catalysis [20, 38], and cysteine residues crucial for the stability may be found in the enzyme’s active site [59]. Also, the disulfide bonds are required for the enzymatic reaction [4]. Qiao and co-workers [60] determined that pullulanase type I from *Exiguobacterium acetylicum* was slightly inhibited by β-mercaptoethanol. In contrast, Pul-SH3 pullulanase type I activity was significantly enhanced by β-mercaptoethanol (1%), and the enzyme retained its initial activity in the presence of 0.02% iodoacetamide (1%) [57]. H_2_O_2_ completely inhibited NP9 pullulanase. H_2_O_2_ oxidizes the methionine residue in the enzyme’s structure and inactivates it [61]. Therefore, it is understood that there are abundant amounts of methionine residues in the structure of NP9 pullulanase. Similar inhibition results were obtained with ApuNP1 pullulanase, and the enzyme was strongly inhibited at 1 and 5% concentrations of H_2_O_2_ [34]. Urea is a denaturant that breaks hydrogen bonds and disrupts protein folds [62, 63]. The slight inhibition of NP9 pullulanase by urea suggests the enzyme is rich in hydrophobic amino acids. Li and co-workers [49] also ascertained that pullulanase type I was inhibited at 2 and 4 M urea concentrations. SH3 pullulanase type I activity was maintained at 1% urea, whereas about 40% was lost at 10% urea [57].

The effect of ionic (CTAB and SDS) and non-ionic (Triton X-100, Tween 20, and Tween 80) detergents on the NP9 pullulanase activity was tested. None of them increased the NP9 pullulanase activity. The enzyme remained moderately stable at both concentrations of these detergent-derived chemicals tested. Similar results were reported in the study by Singh and colleagues [8]. Pullulanase from *B. cereus* ATCC 14579 exhibited approximately 20% residual activity when exposed to 1% SDS. The activity of pullulanase type I from *Metabacillus indicus* increased after 30-min incubation with Triton X-100, Tween 20, and Tween 80. However, the activity of this enzyme decreased to 22.36% in the presence of CTAB, one of the ionic detergents, while it was preserved at 47.11% and 38.9% in the presence of 0.25 and 0.5 mM SDS, respectively [13].

NP9 pullulanase showed moderate stability when exposed to organic solvents such as ethanol, acetone, *n*-butanol, isopropanol, and methanol. If organic solvent molecules replace the water molecules in the enzyme’s structure, the enzyme becomes stable [64]. In the studies of Zafar and collaborators [46], the pullulanase exhibited residual activity levels of 94%, 90%, 75%, 71%, and 69% when exposed to 10% concentrations of methanol, ethanol, acetone, n-butanol, and isopropanol, respectively.

### Effects of NaCl

**Fig. 6:**
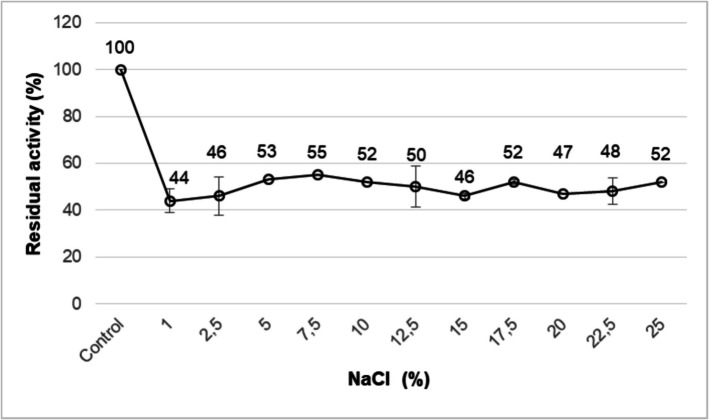
Effect of NaCl on the stability of NP9 pullulanase. Each value is the average of three replicates, and standard error bars are included

The fact that NP9 pullulanase retained almost 50% of its initial activity at 1 to 20% NaCl concentrations indicates that the enzyme is moderately halotolerant **(**Fig. 6**)**. This property shows that the enzyme may be a suitable detergent additive for laundry detergents used in washing with salty groundwater [65]. This stability property of the enzyme is significant in the detergent industry. It can be said that the enzyme that maintains its activity in the presence of this component is also preferable for laundry detergent formulations. Several studies in the literature have determined the effects of various concentrations of NaCl on pullulanase [7, 13, 29].

### Determination of Hydrolytic Properties of NP9 Pullulanase by TLC and HPLC

**Fig. 7:**
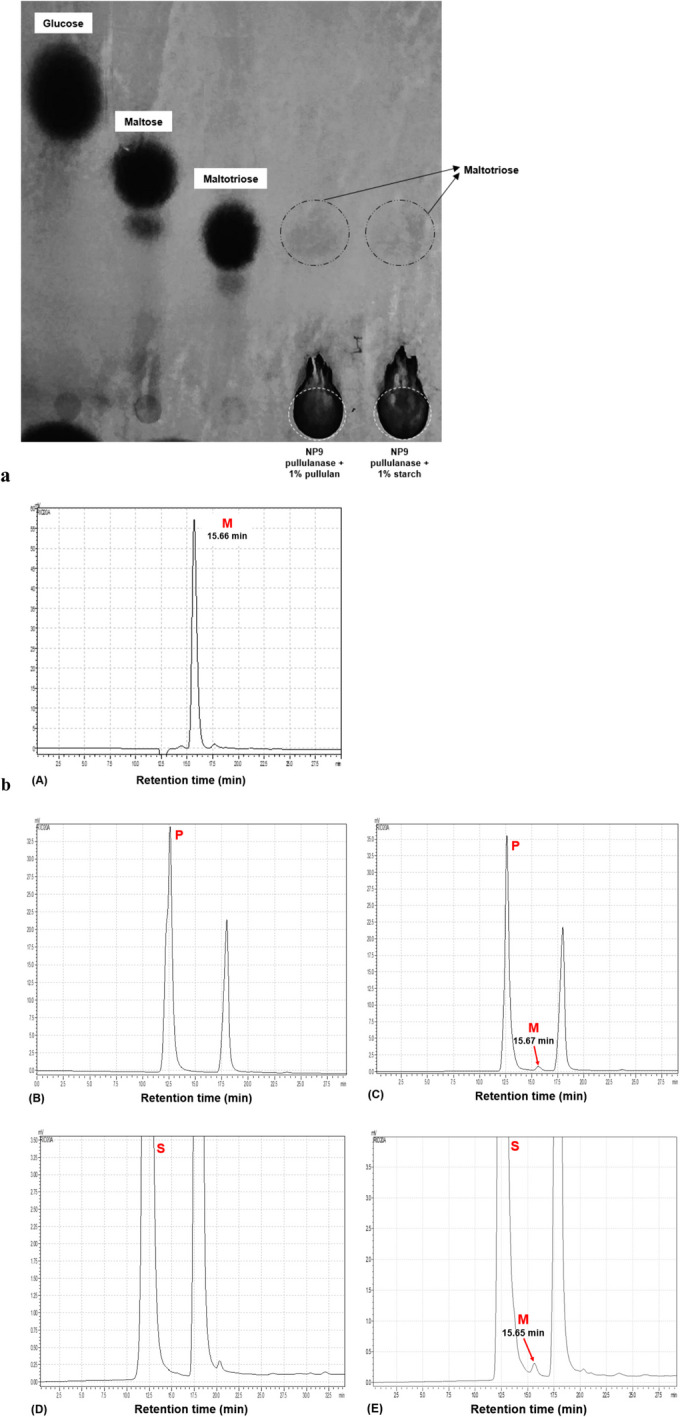
**a** TLC of enzymatic hydrolysis products of pullulan and starch by NP9 pullulanase. Lanes 1, 2, 3, 4, and 5 indicate glucose, maltose, maltotriose, 1% pullulan hydrolysis product, and 1% starch hydrolysis product, respectively. **b** HPLC analysis of products in the hydrolysis of pullulan and starch. (A) Maltotriose standard; (B) pullulan substrate; (C) after incubation of NP9 pullulanase and pullulan; (D) starch substrate; (E) after incubation of NP9 pullulanase and starch. M, maltotriose; P, pullulan; S, starch

The end products of the enzyme–substrate reaction were analyzed by TLC and HPLC. In both analyses, after incubation of NP9 pullulanase with pullulan and starch, the hydrolytic reaction product patterns revealed the conversion of pullulan and starch to maltotriose. According to the data, NP9 pullulanase specifically attacks α−1,6 bonds of the hydrolyzed substrates and can be classified as pullulanase type I. The fact that the enzyme hydrolyzes starch only to maltotriose units, not to glucose and maltose, shows that it affects the α−1,6-glycosidic linkages of the amylopectin unit in starch [1]. As seen in TLC, no glucose and maltose unit spots were detected on the plate in the enzyme reaction with either pullulan or starch (Fig. 7a)**.** In HPLC analysis, the absence of glucose, maltose, isopanose, or panose products (data not shown) supports that the enzyme is involved in the pullulanase class (Fig. 7b). Pullulanases with similar findings in the literature have also been defined as pullulanase type I [1, 4, 23, 47, 50, 66].

### Compatibility Analysis of NP9 Pullulanase

**Fig. 8:**
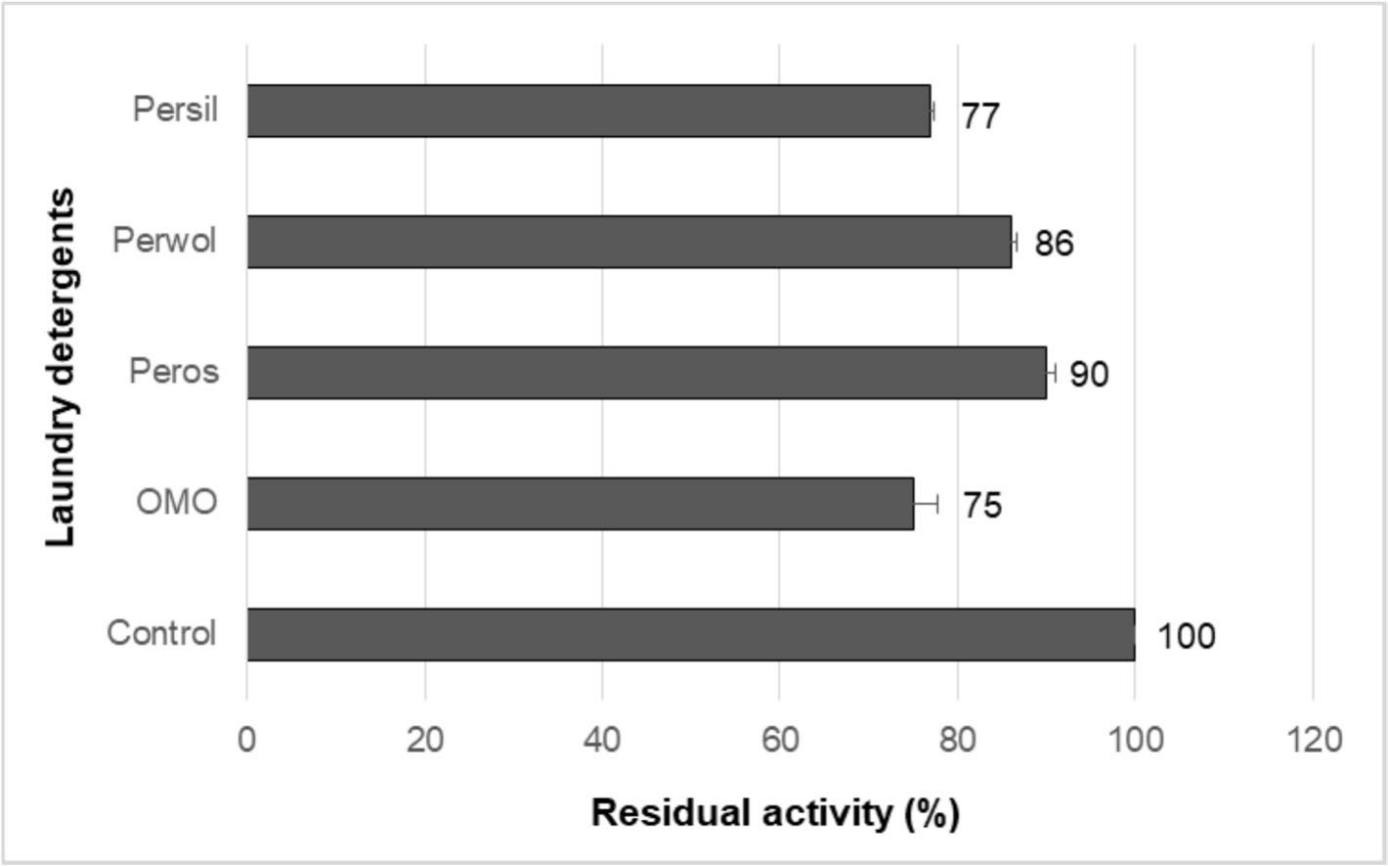
Compatibility analysis of NP9 pullulanase with various commercial liquid laundry detergents. Each value is the average of three replicates, and standard error bars are included

Depending on the stability of NP9 pullulanase, compatibility analysis was performed with various enzyme-based commercial liquid laundry detergents. Among the commercial laundry detergents tested, maximum activity was achieved when Peros (1%) was combined with NP9 pullulanase (5%). As shown in Fig. 8, the enzyme was more than 75% compatible with all tested detergents and maintained its activity by 90% with the “Peros” brand detergent. The enzyme was more adapted and compatible with Peros than other detergents tested. There are some similar studies in the literature with different commercial detergents [35, 46, 57, 67]**.** The strong compatibility of NP9 pullulanase with commercial laundry detergent indicates its potential use in detergent formulations.

### Wash Performance Analysis of NP9 Pullulanase

**Fig. 9:**
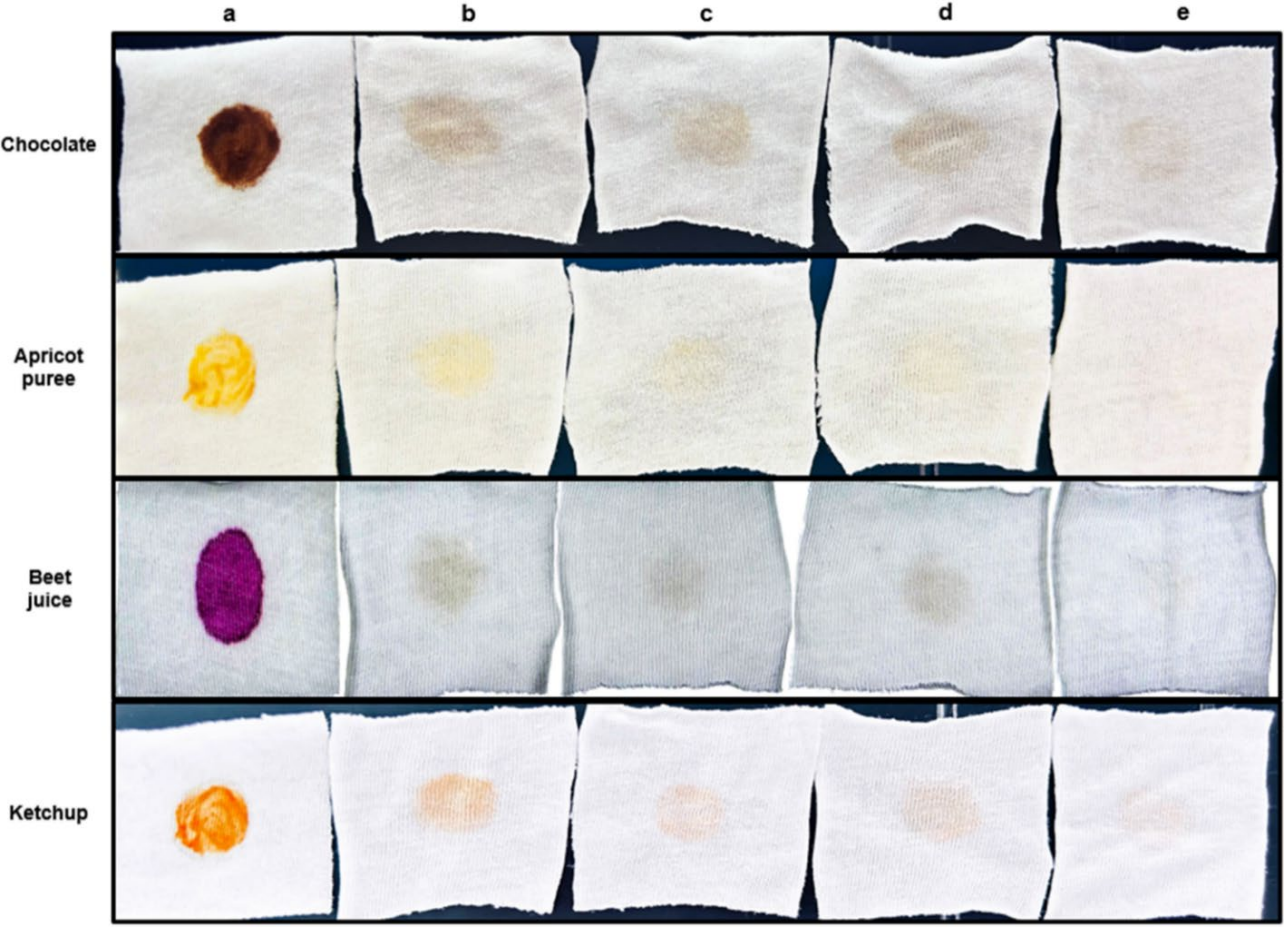
Wash performance analysis of NP9 pullulanase. **a** Untreated stained fabrics; **b** stained cloth pieces + tap water (control); **c** stained cloth pieces + commercial detergent solution; **d** stained cloth pieces + enzyme; **e** stained cloth pieces + enzyme + commercial detergent solution

Images of the starch-based stain removal capacity obtained from the washing performance analysis of NP9 pullulanase are shown in Fig. 9. In the wash performance analysis conducted with all stains, the stain removal capacity in the washing tests where enzyme and detergent were used together was better compared to the control and the washing conditions with only detergent and only enzyme **(**Fig. 9e**)**. Especially according to the washing results of apricot puree and beet juice stains, where enzyme and commercial detergent were used together, almost no stains were observed on the fabrics. According to these results, wash performance analysis with various starch-based stains revealed that NP9 pullulanase could remarkably improve the stain removal capacity of commercial liquid laundry detergents under mild washing conditions. The combined use of the NP9 pullulanase and commercial detergent is more effective in removing stains than washing with commercial detergent alone, as displayed in Fig. 9. Therefore, NP9 pullulanase has significant potential as a detergent bio-additive in liquid laundry detergents suitable for moderate washing conditions, which are generally preferred in washing machines. The NP9 pullulanase is a suitable detergent additive for laundry detergent formulations because it is very effective in removing starchy stains. There are not many quantitative washing performance analysis studies carried out with pullulanases, especially pullulanase type I, in the literature. In this context, our study will contribute to similar research, representing a noteworthy addition to the literature. Dakhmouche Djekrif and co-workers [36] tested the stain removal ability of amylopullulanase on chocolate-jam stain in their work. Their findings indicated that the stain removal performance from the wash analysis, which utilized both the enzyme and detergent, surpassed that of washings conducted with just the detergent or the enzyme. Based on their wash analysis performed at 45 °C, they also noted that the isolated enzyme demonstrated good stability and compatibility with commercial laundry detergents. Additional similar studies have been conducted where enzymes were used with commercial laundry detergents, achieving effective stain removal in comparison to washing with detergent alone [35, 38, 46].

**Fig. 10:**
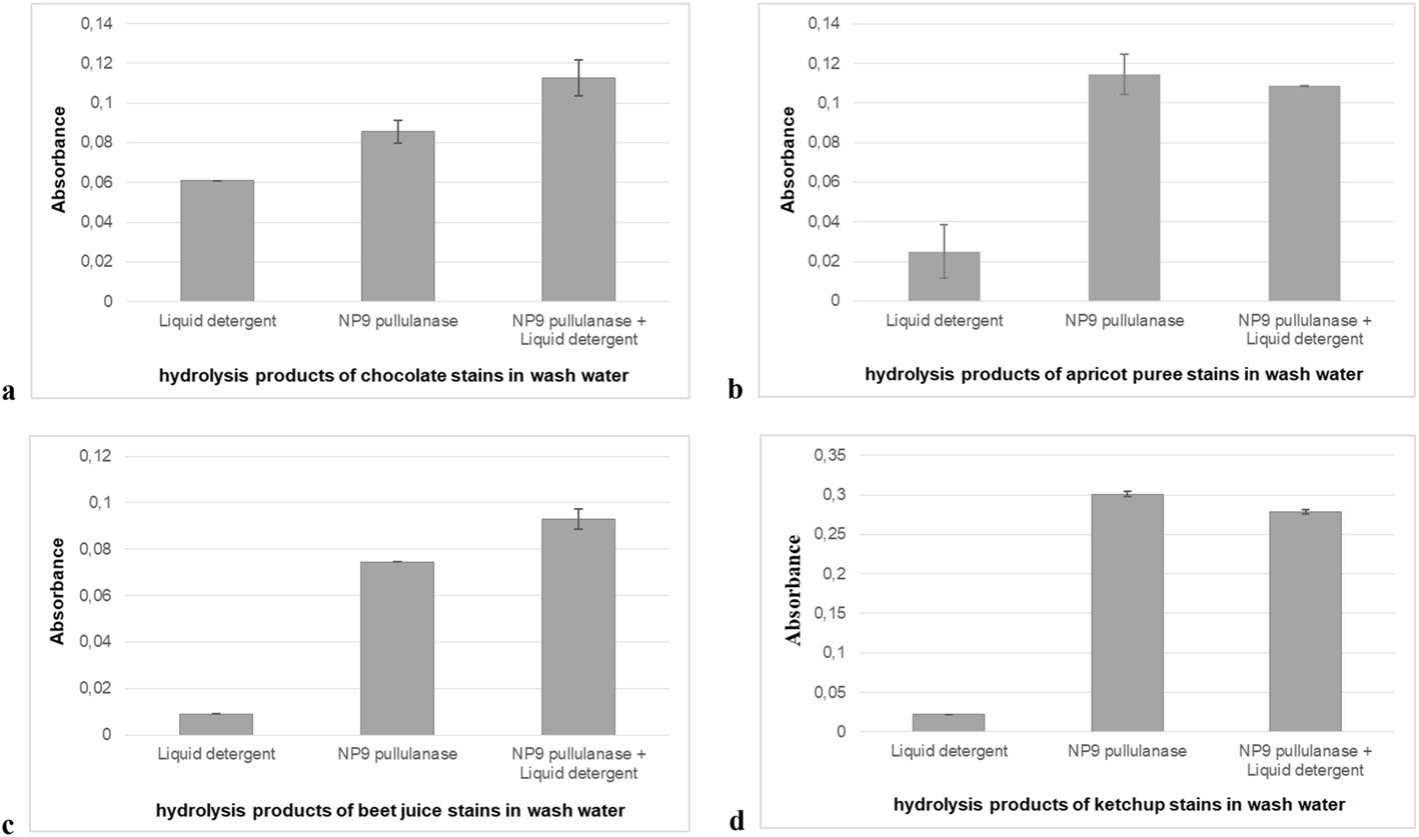
Remained hydrolysis products in the wash water of the starchy stains: **a** chocolate; **b** apricot puree; **c** beet juice; **d** ketchup. Each value is the average of three replicates, and standard error bars are included

In our study, in addition to the washing performance analysis, the “activity analysis from the washing liquid” that we performed is a parameter that is rarely included in the literature [35]. This analysis performed standard enzyme activity at 540 nm to determine the reducing sugar release using the washing liquids obtained from the washing performance analysis performed with stained fabrics **(**Fig. 10**)**. Only the liquid from washing with tap water was used as a blank. After the incubation period, washing water samples were collected from the beakers containing the stained fabrics in which the washing was carried out, and standard activity analyses were conducted with each sample using the DNS method. The activity values indicate the amount of reducing sugar passed into the washing water. High activity in the washing water suggests that stain removal occurs more in that washing environment. This analysis is also compatible with the stain removal capacity findings obtained as a result of the washing performance analysis and can be considered a verification and proof of the washing performance analysis results.

## Conclusion

In the present study, *Bacillus cereus* strain NP9, a bacterium with extracellular pullulanase activity, was isolated. The isolated novel pullulanase was characterized, and the applicability of this enzyme in the detergent industry was investigated. The enzyme’s significant stability in the pH range of 5.0–11.0 and temperature between 25 and 65 °C, its ability to hydrolyze alpha-1,6 glucosidic bonds of pullulan and starch, and its non-Ca^2+^ dependent metalloenzyme nature make NP9 pullulanase preferable in various industrial processes. Results revealed that using NP9 pullulanase enhances the removal of starch-based stains from cotton cloths when combined with commercial laundry detergent, compared to using either pullulanase or detergent alone. Including certain microbial enzymes in detergents significantly contributes to developing formulas that benefit human health, skin, and the environment. Thus, incorporating NP9 pullulanase, characterized in our study, into existing detergent formulations presents a remarkable opportunity for the detergent industry. NP9 pullulanase may be used as an additive in liquid laundry detergent, working under moderate conditions. Additionally, this enzyme possesses valuable physicochemical properties that can be utilized in starch processing (for granular starch hydrolysis), the textile industry, oligosaccharide production, and toothpaste production (as a dental plaque control agent).

## Data Availability

All data from the mentioned study are included in this article.
